# Association between serum uric acid and triglyceride-glucose index in children and adolescents with short stature

**DOI:** 10.1038/s41598-023-40972-2

**Published:** 2023-08-21

**Authors:** Qianqian Zhao, Mei Zhang, Yuntian Chu, Bo Ban

**Affiliations:** 1 School of Medicine, Qingdao University, Qingdao, 266071 Shandong People’s Republic of China; 2 Department of Endocrinology, Affiliated Hospital of Jining Medical University, Jining Medical University, 89 Guhuai Road, Jining, 272029 Shandong People’s Republic of China; 3 Chinese Research Center for Behavior Medicine in Growth and Development, 89 Guhuai Road, Jining, 272029 Shandong People’s Republic of China; 4 National Telemedicine Center of China, The First Affiliated Hospital of Zhengzhou University, Zhengzhou, 450099 Henan People’s Republic of China

**Keywords:** Dyslipidaemias, Growth disorders

## Abstract

The aim of this study was to explore the relationship between serum uric acid (SUA) and the triglyceride-glucose (TyG) index, which is a more effective indicator of insulin resistance. The study participants included 1700 children and adolescents with short stature who were recruited at the Affiliated Hospital of Jining Medical University in China between March 2013 and April 2021. A positive association between SUA levels and the TyG index was detected by univariate analysis (*p* < 0.001). Furthermore, a nonlinear relationship was detected between SUA and the TyG index, whose point was 6.55 mg/dL. There was a positive association between SUA and the TyG index when the SUA level was greater than 6.55 mg/dL (β 0.17, 95% CI: 0.07, 0.27; *P* < 0.001). However, we did not observe a significant relationship between SUA and the TyG index when the SUA level was less than 6.55 mg/dL (β 0.02, 95% CI: − 0.01, 0.05; *P* = 0.091). In addition, a stratified analysis was performed to appraise changes in this relationship for different sexes. The relationship between SUA and the TyG index in males and females is consistent with that in the general population, showing a nonlinear relationship. However, the inflection points of SUA level were significantly higher in males than in females, and the inflection points were approximately 6.72 and 5.88 mg/dL, respectively. This study revealed a nonlinear relationship between SUA and the TyG index in children with short stature. The nonlinear relationship remained in gender stratification analysis, but the inflection point of SUA level was higher in men. Further studies are needed to establish a causal relationship between SUA levels and the TyG index in children with short stature.

## Introduction

Serum uric acid (SUA) is the end product of purine metabolism, which mainly involves the decomposition of nucleic acids and other purine compounds in cells and purines in food through the action of enzymes^1^. It has been recognized that SUA has a strong antioxidant effect located extracellularly and can scavenge oxygen free radicals generated by oxidative stress and prevent oxidative damage^2^. There are many factors influencing SUA levels, including a variety of internal and external factors, such as renal and extrarenal excretion of SUA, genetics and diet^3^. However, there is evidence supporting the involvement of elevated SUA levels in the progression of a variety of diseases, including hypertension, type 2 diabetes, coronary heart disease, dyslipidemia, metabolic syndrome and renal complications^4–6^. Insulin resistance plays a key pathological role in these metabolic diseases^7^. Recently, increasing evidence has shown a close relationship between SUA and insulin resistance (IR). While controversy exists regarding the causal relationship between them in previous reports^8–12^, numerous studies have indicated that hyperuricemia may be an independent risk factor for IR^8,10^.

IR has been assessed by well-established methods, such as the hyperinsulinemic euglycemic glucose clamp (HEGC), known as the "gold standard" for the assessment of insulin resistance. However, it is difficult to carry out in clinical practice because of its frequent blood collection, complicated indexes and high cost^13^. In recent years, the triglyceride glucose (TyG) index, namely, the product of triglyceride (TG) and fasting plasma glucose (FPG), has been shown to be more effective in detecting IR due to its good correlation with HOMA-IR^14^. Hyperuricemia can induce IR by promoting oxidative stress, inducing inflammation, and disrupting insulin signaling pathways^8^. An observational study of 5,012 healthy adolescents followed for 15 years supports the use of SUA concentration as a simple indicator for assessing the future occurrence of type 2 diabetes and IR^12^. A recent longitudinal cohort study that included 8,543 participants showed that elevated SUA levels precede IR, highlighting the potential relationship between hyperuricemia and IR^10^.

A strong correlation between IR and the risk of developing cardiovascular disease (CVD) has been established. Some studies have provided evidence that short stature is a risk factor associated with CVD^15^. Furthermore, recent evidence suggests that height and IR are also negatively correlated^16^, suggesting that individuals with short stature have a significantly increased risk of developing CVD and IR. Additionally, a longitudinal study conducted in children with idiopathic short stature revealed a correlation between SUA levels and height standard deviation score (SDS)^17^. Furthermore, short stature in childhood is associated with health status in adulthood^18^. Therefore, it is necessary to pay attention to SUA status in children with short stature as a risk factor for IR. However, studies on the relationship between SUA and the TyG index in children, especially children with short stature, have been limited. We hypothesize that there is an association between SUA levels and the TyG index in children with short stature. Therefore, the purpose of this study was to investigate the relationship between SUA levels and the TyG index in children with short stature.

## Results

### Characteristics of the study population

The baseline characteristics of all study participants are summarized in Table 1. The present study included 1,700 participants. Among the participants, approximately 66.6% were male, and the mean age of this cohort was 10.1 ± 3.7 years. The mean height SDS was − 2.7 ± 0.7 for all subjects, and the mean height SDS was also − 2.7 ± 0.7 for both males and females, with no significant difference (*P* = 0.665). In the study population, the majority of the children, 1178 (69.3%), were prepubescent. The mean TyG index and SUA level were 7.8 ± 0.4 and 4.5 ± 1.2 mg/dL, respectively. The TyG index and SUA level in males were significantly higher than those in females (both *P* < 0.05). In the present study, the overall prevalence of hyperuricemia was 17.0%, with prevalences of 19.1 and 12.8% in males and females, respectively.

**Table 1:** Clinical and biochemical characteristics.

Variables	All	Male	Female	*P* value
Number	1700	1132	568	–
Age (years)	10.1 ± 3.7	10.5 ± 3.7	9.2 ± 3.3	< 0.001
Height (cm)	124.3 ± 18.2	126.9 ± 18.7	119.2 ± 16.1	< 0.001
Height SDS	− 2.7 ± 0.7	− 2.7 ± 0.7	− 2.7 ± 0.7	0.665
Body weight (kg)	27.1 ± 11.1	28.7 ± 11.8	23.9 ± 8.9	< 0.001
BMI (kg/m2)	16.8 ± 3.1	17.0 ± 3.2	16.2 ± 2.7	< 0.001
Birth length (cm)	49.7 ± 2.6	49.8 ± 2.6	49.6 ± 2.5	0.278
Birth weight (kg)	3.1 ± 0.5	3.2 ± 0.5	3.0 ± 0.5	< 0.001
SBP (mmHg)	105.5 ± 12.1	106.8 ± 12.2	102.7 ± 11.1	< 0.001
DBP (mmHg)	62.4 ± 8.7	62.7 ± 8.7	61.7 ± 8.5	0.061
IGF-1 (ng/ml)	163.0 (95.8–243.0)	167.0 (90.9–252.0)	159.5 (105.8–232.0)	0.604
IGF-1 SDS	− 1.0 (− 1.7–0.1)	− 0.9 (− 1.6–0.1)	− 1.1 (− 2.0–0.4)	< 0.001
Cr (umol/L)	39.3 ± 9.6	40.6 ± 9.8	36.6 ± 8.5	< 0.001
BUN (mmol/L)	4.6 ± 1.1	4.7 ± 1.1	4.3 ± 1.0	< 0.001
SUA (mg/dL)	4.5 ± 1.2	4.6 ± 1.2	4.3 ± 1.0	< 0.001
FPG (mmol/L)	4.8 ± 0.6	4.8 ± 0.6	4.7 ± 0.6	< 0.001
TG (mmol/L)	0.7 (0.5–0.9)	0.7 (0.5–0.9)	0.7 (0.5–0.8)	0.027
TC (mmol/L)	3.9 ± 0.7	3.8 ± 0.7	4.0 ± 0.8	< 0.001
HDL (mmol/L)	1.4 ± 0.3	1.4 ± 0.3	1.4 ± 0.3	0.197
LDL (mmol/L)	2.1 ± 0.6	2.1 ± 0.6	2.2 ± 0.6	< 0.001
TyG index	7.8 ± 0.4	7.9 ± 0.5	7.8 ± 0.4	0.011
Pubertal stage				0.409
In prepuberty (%)	1178 (69.3%)	777 (68.6%)	401 (70.6%)	
In puberty (%)	522 (30.7%)	355 (31.4%)	167 (29.4%)	
Hyperuricemia				0.003
No	1198 (83.0%)	776 (80.9%)	422 (87.2%)	
Yes	245 (17.0%)	183 (19.1%)	62 (12.8%)	

*Height SDS* height standard deviation scores, *BMI* body mass index, *SBP* systolic blood pressure, *DBP* diastolic blood pressure, *IGF-1 SDS* insulin like growth factor-1 standard deviation scores, *Cr* creatinine, *BUN* blood urea nitrogen, *SUA* serumuric acid, *FPG* fasting plasma glucose, *TG* triglyceride, *TC* total cholesterol, *HDL-C* high density lipoprotein-cholesterol, *LDL-C* low density lipoprotein cholesterol, *TyG index* triglyceride glucose index.

Continuous variables are presented as the mean ± standard deviation or median (interquartile range). Categorical variables are displayed as number (percentage).

### Factors associated with SUA in the subjects

The results of the univariate analyses of the relationships between clinical parameters and TyG index are presented in Table 2. The results indicated that age, weight, body mass index (BMI), systolic blood pressure (SBP), insulin-like growth factor-1 standard deviation scores (IGF-1 SDS), creatinine (Cr), SUA, total cholesterol (TC), low density lipoprotein cholesterol (LDL-C) and pubertal stage were positively related to the TyG index, whereas blood urea nitrogen (BUN) and high density lipoprotein-cholesterol (HDL-C) were negatively associated with the TyG index (all *P* < 0.001). However, the results showed that the relationships between the TyG index and height SDS, birth length, birth weight, and diastolic blood pressure (DBP) were not significant in the present study (all *P* > 0.05). In addition, the relationship between clinical parameters and the TyG index in males and females was consistent with that in all study subjects.

**Table 2:** Association between the TyG index and different variables.

Variables	All		Male		Female	
	β (95% CI)	*P* value	β (95% CI)	*P* value	β (95% CI)	*P* value
Age (years)	0.02 (0.02, 0.03)	< 0.001	0.03 (0.02, 0.03)	< 0.001	0.02 (0.01, 0.03)	0.019
Height SDS	0.02 (− 0.01, 0.04)	0.289	0.03 (− 0.01, 0.06)	0.167	− 0.01 (− 0.05, 0.04)	0.845
Body weight (kg)	0.01 (0.01, 0.01)	< 0.001	0.01 (0.01, 0.01)	< 0.001	0.01 (0.01, 0.01)	0.001
BMI (kg/m^2^)	0.04 (0.03, 0.05)	< 0.001	0.04 (0.03, 0.05)	< 0.001	0.02 (0.01, 0.04)	0.013
Birth length (cm)	0.05 (− 0.01, 0.02)	0.465	0.01 (− 0.01, 0.02)	0.611	0.01 (− 0.02, 0.03)	0.626
Birth weight (kg)	0.02 (− 0.03, 0.07)	0.408	0.01 (− 0.04, 0.07)	0.642	0.02 (− 0.07, 0.11)	0.692
SBP (mmHg)	0.01 (0.01, 0.01)	< 0.001	0.01 (0.01, 0.01)	< 0.001	0.01 (− 0.01, 0.01)	0.297
DBP (mmHg)	0.01 (− 0.01, 0.01)	0.162	0.01 (− 0.01, 0.01)	0.066	− 0.01 (− 0.01, 0.01)	0.567
IGF-1 SDS	0.04 (0.02, 0.06)	< 0.001	0.05 (0.02, 0.07)	< 0.001	0.02 (− 0.01, 0.04)	0.271
Cr (umol/L)	0.01 (0.01, 0.01)	< 0.001	0.01 (0.01, 0.01)	< 0.001	0.01 (0.01, 0.01)	< 0.001
BUN (mmol/L)	− 0.04 (− 0.06, − 0.01)	< 0.001	− 0.04 (− 0.07, − 0.01)	0.003	− 0.05 (− 0.09, − 0.01)	0.002
SUA (mg/dL)	0.05 (0.03, 0.07)	< 0.001	0.05 (0.02, 0.07)	< 0.001	0.05 (0.01, 0.09)	0.012
TC (mmol/L)	0.09 (0.06, 0.12)	< 0.001	0.11 (0.06, 0.15)	< 0.001	0.08 (0.03, 0.13)	0.001
HDL (mmol/L)	− 0.35 (− 0.43, − 0.27)	< 0.001	− 0.40 (− 0.50, − 0.31)	< 0.001	− 0.23 (− 0.36, − 0.10)	< 0.001
LDL (mmol/L)	0.11 (0.07, 0.15)	< 0.001	0.14 (0.08, 0.19)	< 0.001	0.08 (0.02, 0.14)	0.014
Pubertal stage						
In prepuberty (%)	Reference				Reference	
In puberty (%)	0.18 (0.13, 0.23)	< 0.001	0.17 (0.11, 0.24)	< 0.001	0.18 (0.10, 0.27)	< 0.001

*Height SDS* height standard deviation scores, *BMI* body mass index standard deviation scores, *SBP* systolic blood pressure, *DBP* diastolic blood pressure, *IGF-1 SDS* insulin like growth factor-1 standard deviation scores, *Cr* creatinine, *BUN* blood urea nitrogen, *SUA* serumuric acid, *TC* total cholesterol, *HDL-C* high density lipoprotein-cholesterol, *LDL-C* low density lipoprotein cholesterol, *TyG index* triglyceride glucose index.

*P* < 0.05 is considered to be statistically significant.

### The results of nonlinearity of the TyG index and SUA

**Figure 1:**
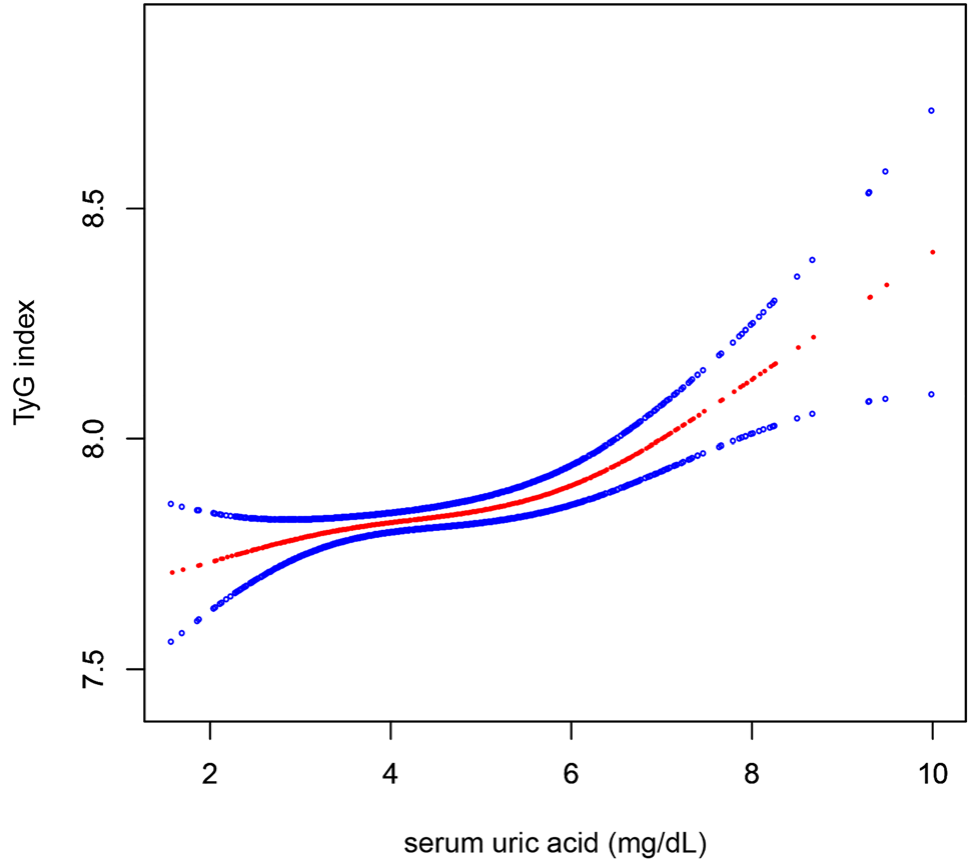
Smooth curve fitting of the relationship between SUA and the TyG index. Adjustment variables: age, sex, BMI, SBP, IGF-1, Cr, BUN, TC, pubertal stage. BMI: body mass index, SBP: systolic blood pressure; IGF-1: insulin like growth factor-1; Cr creatinine; BUN blood urea nitrogen; TC: total cholesterol; SUA serumuric acid; TyG index: triglyceride glucose index.

To explore the nonlinear relationship between SUA and the TyG index, smooth curve fitting was carried out. After adjusting for potential confounding factors, including age, sex, BMI, SBP, IGF-1, Cr, BUN, TC and pubertal stage, the results showed a significant nonlinear relationship between SUA and the TyG index. An inflection point was observed in this study, indicating different relationships between SUA and the TyG index depending on the SUA level (Fig. 1). We further conducted two piecewise linear regressions and identified the inflection point of the SUA level as 6.55 mg/dL (Table 3). When the SUA level was greater than 6.55 mg/dL, the SUA and TyG index appeared to have a significantly positive relationship; as the SUA level rose, the TyG index gradually increased (β 0.17, 95% CI: 0.07, 0.27; *P* < 0.001). However, when the SUA level was less than 6.55 mg/dL, the relationship between SUA and the TyG index was not significant (β 0.02, 95% CI: − 0.01, 0.05; *P* = 0.091). In addition, the log likelihood ratio test was carried out to compare the single linear regression model and the two piecewise linear models to determine which is more suitable to represent the relationship between SUA and the TyG index. The results show that the two piecewise linear models can better represent the real relationship between SUA and the TyG index (*P* = 0.008).

**Table 3:** Threshold effect analysis for the relationship between SUA and the TyG index.

Models	TyG index	
	Adjusted β (95%CI)	*P* value
Model I		
One line slope	0.04 (0.02, 0.06)	< 0.001
Model II		
Turning point (K)	6.55	
< K slope 1	0.02 (-0.01, 0.05)	0.091
> K slope 2	0.17 (0.07, 0.27)	< 0.001
LRT test	0.008	

Model I, linear analysis; Model II, non-linear analysis. LRT test, Logarithmic likelihood ratio test. (*p* value < 0.05 means Model II is significantly different from Model I, which indicates a non-linear relationship); Adjustment variables: age, sex, BMI, SBP, IGF-1, Cr, BUN, TC, pubertal stage.

*BMI* body mass index, *SBP* systolic blood pressure, *IGF-1* insulin like growth factor-1, *Cr* creatinine, *BUN* blood urea nitrogen, *TC* total cholesterol, *SUA* serumuric acid, *TyG index* triglyceride glucose index.

*P* < 0.05 is considered to be statistically significant.

**Figure 2:**
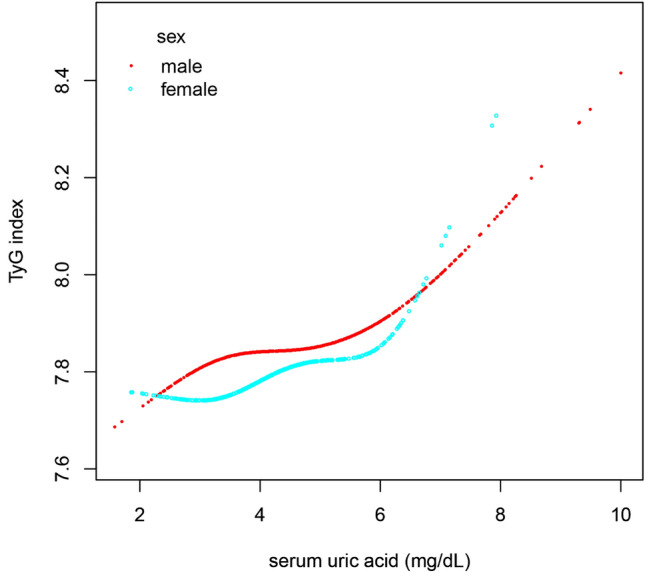
Smooth curve fitting of the relationship between SUA and the TyG index in different sexes. Adjustment variables: age, BMI, SBP, IGF-1, Cr, BUN, TC, pubertal stage. BMI: body mass index, SBP: systolic blood pressure; IGF-1: insulin like growth factor-1; Cr creatinine; BUN blood urea nitrogen; TC: total cholesterol; SUA serumuric acid; TyG index: triglyceride glucose index.

A stratified analysis of the relationship between SUA and the TyG index in different sexes was performed with a fit curve, as displayed in Fig. 2, and piecewise linear regression, as displayed in Table 4. The relationship between SUA and the TyG index in males and females is consistent with that in the general population, showing a nonlinear relationship (Fig. 2). However, the inflection points of SUA level were significantly higher in males than in females, and the inflection points were approximately 6.72 and 5.88 mg/dL, respectively (Table 4). In males, at SUA levels higher than 6.72 mg/dL, the TyG index gradually increased with increasing SUA levels (β = 0.19, 95% CI: 0.07, 0.31; *P* = 0.001), but no association was found when SUA levels were less than 6.72 mg/dL (β = 0.03, 95% CI: − 0.01, 0.06; *P* = 0.061). Moreover, a similar relationship was found among females. The TyG index increased as SUA levels increased when SUA levels were greater than 5.88 mg/dL (β = 0.27, 95% CI: 0.07, 0.47; *P* = 0.009), yet no significant relationship was observed at lower SUA concentrations (β = 0.02, 95% CI: − 0.03, 0.07; *P* = 0.386).

**Table 4:** Sex-specific threshold effect analysis for the relationship between SUA and the TyG index.

Models	Male		Female	
	Adjusted β (95%CI)	*P* value	Adjusted β (95%CI)	*P* value
Model I				
One line slope	0.04 (0.02, 0.06)	0.002	0.05 (0.01, 0.08)	0.014
Model II				
Turning point (K)	6.72		5.88	
< K slope 1	0.03 (− 0.01, 0.06)	0.061	0.02 (− 0.03, 0.07)	0.386
> K slope 2	0.19 (0.07, 0.31)	0.001	0.27 (0.07, 0.47)	0.009
LRT test	0.018	0.031		

Model I, linear analysis; Model II, non-linear analysis. LRT test, Logarithmic likelihood ratio test. (*p* value < 0.05 means Model II is significantly different from Model I, which indicates a non-linear relationship); Adjustment variables: age, BMI, SBP, IGF-1, Cr, BUN, TC, pubertal stage.

*BMI* body mass index, *SBP* systolic blood pressure, *IGF-1* insulin like growth factor-1, *Cr* creatinine, *BUN* blood urea nitrogen, *TC* total cholesterol, *SUA* serumuric acid, *TyG index* triglyceride glucose index.

*P* < 0.05 is considered to be statistically significant.

## Discussion

The primary finding of the study was that there was a significant positive relationship between SUA and the TyG index in children with short stature. Interestingly, a nonlinear relationship between SUA and the TyG index was identified in children with short stature. The results showed that the SUA level turning point was 6.55 mg/dL, and there was a positive relationship between SUA and the TyG index when the SUA level was greater than 6.55 mg/dL, whereas a significant relationship between SUA and the TyG index was not observed when the SUA level was lower than 6.55 mg/dL. In addition, a stratified analysis of the relationship between SUA and the TyG index in different sexes showed that the relationship between SUA and the TyG index in males and females was consistent with that in the general population. However, the inflection points of SUA level were significantly higher in males than in females, and the inflection points were approximately 6.72 and 5.88 mg/dL, respectively.

In this study, a linear regression model was performed and revealed a positive association between SUA and the TyG index in short children, even after adjusting for confounding factors. To our knowledge, this study is the first to reveal an association between SUA and the TyG index in short children, which is consistent with findings in adults^19,20^. Feitosa RS et al. explored the relationship between SUA and IR in women from northeastern Brazil, a study that also assessed IR using the TyG index, and the results of the study found that the TyG index was significantly higher in the hyperuricemic population than in the non-hyperuricemic population, suggesting that hyperuricemia is a risk factor for IR^19^. Moreover, In a group of Framingham Heart Study Generation 3 participants without significant CVD, diabetes, obesity, or hypertension, SUA showed a significant association with IR^20^. In addition, a recent study has shown that SUA is associated with cardiometabolic conditions such as IR and visceral adipose tissue (VAT) accumulation, and that elevated SUA plays a mediating role in the bidirectional relationship between IR and VAT accumulation^21^. The study found that there may be similar results in terms of SUA and the TyG index between short stature children and the general population. This may indicate that short stature children have some metabolic changes that are similar to those of children and adults of normal stature. However, further study is needed to determine the exact relationship between SUA and the TyG index in short stature children compared to children and adults of normal stature. Other factors, such as dietary habits, genetic factors, and lifestyle, may also influence these results. In conclusion, when evaluating the metabolic risks of short stature children, SUA and the TyG index may be considered relevant indicators. Further research can help us better understand the metabolic characteristics of short children and develop appropriate prevention and treatment strategies.

The causal relationship between hyperuricemia and IR is controversial. Based on pathological and physiological research, it is believed that hyperuricemia and IR may mutually influence each other. SUA can lead to IR by reducing the bioavailability of nitric oxide (NO) and generating mitochondrial oxidative stress^22,23^. Conversely, one of the primary mechanisms through which IR inhibits SUA excretion is by increasing renal tubular sodium reabsorption, resulting in hyperuricemia^24^. Although many studies have observed the impact of the TyG index on hyperuricemia, current knowledge supports the idea of a vice versa causality^8,10^. This study describes the relationship between SUA and the TyG index in children with short stature. Interestingly, we further observed the nonlinear relationship between SUA and the TyG index through smooth curve fitting. This indicates that SUA and the TyG index are not simply linear associations, and there may be a threshold effect; that is, when SUA reaches a certain level, the association between SUA and the TyG index is significant. By analyzing the threshold saturation effect, we find that the inflection point of SUA levels is 6.55 mg/dL. The nonlinear relationship between SUA and the TyG index may be because SUA starts to have a significant impact on metabolic processes only after reaching a certain threshold. At lower levels of SUA, it may not have a significant effect on IR and metabolic indicators. However, once SUA levels exceed 6.55 mg/dL, a threshold effect may occur, where the increase in SUA begins to significantly affect insulin sensitivity and metabolic status, resulting in changes in the TyG index. Previous studies have shown that there is not a simple linear relationship between uric acid and a variety of metabolic indicators but a nonlinear relationship; that is, there may be a threshold effect^25–28^. Recent studies have revealed that the threshold at which SUA exerts its effects may vary in different situations. A study found that SUA levels ≥ 4.7 mg/dL are associated with all-cause mortality, while SUA levels ≥ 5.6 mg/dL are associated with cardiovascular mortality^4^. In addition, when SUA levels reach 5.1 mg/dL in females and 5.6 mg/dL in males, there is a stronger association between SUA and atherogenic lipoproteins (LDL-C and non-HDL-C)^5^. This threshold effect may be related to the biological function of SUA. Hyperuricemia is associated with cardiovascular disease, IR, and metabolic disorders^4,8,29,30^. When uric acid levels surpass a certain threshold, they may have a more pronounced effect on these metabolic processes, leading to a significant relationship between SUA and the TyG index.

In the present study, differences were found in the relationship between SUA and the TyG index between males and females, with a higher SUA threshold in males. This is consistent with the findings of a previous nationwide cohort study that investigated the association between SUA levels and long-term mortality related to metabolic dysfunction-associated fatty liver disease. In males, there was an elevated risk of CVD mortality associated with higher SUA levels when SUA exceeded 6.7 mg/dL. For females, a significant positive correlation between SUA levels and CVD risk and cancer mortality was observed only when SUA exceeded 5.5 mg/dL^25^. In general, in both adults and children, males tend to have higher SUA levels than females^31^. This difference is likely due to the distinct roles of sex hormones and a higher muscle mass in males^32^. Testosterone, which is present at higher levels in males, has been found to increase SUA production and decrease its excretion. On the other hand, estrogen, which is predominant in females, has a uricosuric effect, promoting the excretion of uric acid from the body. These hormonal differences contribute to the higher SUA levels observed in males^33^. Additionally, skeletal muscle mass and strength play a role in SUA metabolism. Skeletal muscle is a major site of purine metabolism, and higher muscle mass in males leads to increased purine turnover and SUA production^34^. This, combined with higher testosterone levels, contributes to the higher SUA levels in males.

There are several limitations of the study. The first is due to the cross-sectional nature of the study. The results of this study can only show that there is an association between SUA and the TyG index, and the causal relationship of this association needs to be confirmed in further longitudinal studies. Second, this study revealed a nonlinear relationship between SUA and the TyG index in Chinese children with short stature. However, we cannot conclude that our research results are applicable to people of different populations and races. Third, this study is a cross-sectional analysis of a cohort of individuals with short stature and does not include children with normal height. In future studies, the influence of height, hormonal changes, and nutritional status on SUA levels can be explored. Finally, consistent with other observational studies, although we included many covariates in our model, unrecorded residual confounding affecting the relationship between SUA levels and the TyG index may bias our findings. In view of this, larger-scale studies including more covariates are needed to confirm the relationship between SUA and the TyG index.

In conclusion, the present study showed a positive association between SUA and the TyG index, and the level of the TyG index increased with increasing SUA. Most importantly, our study revealed a nonlinear relationship between SUA and the TyG index. The nonlinear relationship remained in gender stratification analysis, but the inflection point of SUA level was higher in men. However, our study does not support a causal role of SUA on IR among children with short stature, and the possible mechanism would require further clarification.

## Methods

### Participants

The present study was an observational cross-sectional study. The study population consisted of patients who were admitted to the Department of Endocrinology, Genetics and Metabolism, Affiliated Hospital of Jining Medical University from March 2013 to April 2021 due to short stature. They are part of the GDDSD study (Growth and Development Diseases in Shandong Province: a cohort follow-up study, http://www.chictr.org.cn, ChiCTR1900026510). A total of 1700 subjects (1132 males and 568 females) with a mean age of 10.1 ± 3.7 years were recruited. The subjects in this study met the following inclusion criteria: children and adolescents with an age range of 3–18 years and height more than two standard deviations (SDs) lower than the average of individuals of the same race, age, and sex. Exclusion criteria included patients who were treated with diuretics and other cardiovascular drugs and patients with chronic diseases, skeletal dysplasia, thyroid dysfunction, or chromosomal abnormalities such as Turner syndrome.

### Ethics

The study protocol was approved by the Human Research Ethics Committee of Affiliated Hospital of Jining Medical University, and all methods were performed in accordance with the guidelines of the Declaration of Helsinki. Written informed consent was obtained from their parents.

### Clinical and laboratory assessments

Height and weight were measured by a stadiometer and electronic scale, respectively. Weight was quantified to the nearest 0.1 kg with subjects wearing light clothing, and height was quantified to the nearest 0.1 cm after the subjects had removed their shoes. BMI was calculated by dividing weight by height squared. Blood pressure was measured 3 times in a sitting position by trained nurses using an HBP 1300 electronic sphygmomanometer (Omron, Dalian, Japan), and the average of the three measurements was used for statistical analysis. Pubertal stage was assessed by physical examination according to Tanner stage^35^. Laboratory examination was performed after overnight fasting for at least 8 h, and the parameters assessed included IGF-1, Cr, BUN, SUA, FPG, TG, TC, HDL-C and LDL-C. The IGF-1 SDS was calculated using the reference value of healthy children of the same age and sex^36^. The TyG index was calculated as ln [fasting TG (mg/dL) × FPG (mg/dL)/2]^37^. Due to the lack of previous standards for hyperuricemia in children and adolescents, we defined hyperuricemia based on the previously reported threshold of 5.5 mg/dL^38,39^.

### Statistical analysis

Data are presented as the mean and SD or median and interquartile range for continuous variables and percentage for categorical variables. We performed a simple linear regression analysis to estimate whether SUA and other indicators were related to the TyG index. Smooth curve fitting was further carried out to explore the nonlinear relationship between SUA and the TyG index. Finally, the linear relationship and threshold association of SUA and the TyG index were estimated by multiple regression analysis and multiple piecewise linear regression, respectively. A log likelihood ratio test was performed to compare the single linear regression model and two piecewise linear models. Statistical significance was indicated by a two-sided *P* value < 0.05. Statistical analysis was performed with R 3.6.1 (https://www.R-project.org) and EmpowerStats (https://www.empowerstats.com; X&Y Solutions, Inc).

### Supplementary Information

Supplementary Information.

## Data Availability

All data generated or analyzed during this study are included in this published article and its supplementary information files.
